# Effect of Printing Parameters on the Dynamic Characteristics of Additively Manufactured ABS Beams: An Experimental Modal Analysis and Response Surface Methodology

**DOI:** 10.3390/polym17121615

**Published:** 2025-06-10

**Authors:** Hilal Doğanay Kati, Feiyang He, Muhammad Khan, Hakan Gökdağ, Yousef Lafi A. Alshammari

**Affiliations:** 1 Centre for Life-Cycle Engineering and Management, Faculty of Engineering and Applied Sciences, Cranfield University, Bedford MK43 0AL, UK; hilal.doganay@cranfield.ac.uk (H.D.K.); y.l.alshammari@cranfield.ac.uk (Y.L.A.A.); 2 Faculty of Engineering and Natural Sciences, Department of Mechanical Engineering, Bursa Technical University, Bursa 16310, Turkey; hakan.gokdag@btu.edu.tr; 3 Mechanical Engineering Department, Engineering College, Northern Border University, King Fahad Road, Arar 92341, Saudi Arabia

**Keywords:** experimental modal analysis, fused deposition modelling, response surface method, damping ratio, natural frequency, ABS

## Abstract

This study investigates the dynamic characteristics of three-dimensional (3D) printed acrylonitrile butadiene styrene (ABS) cantilever beams using Experimental Modal Analysis (EMA). The effects of Fused Deposition Modelling (FDM) process parameters—specifically infill pattern, infill density, nozzle size, and raster angle—on the natural frequency, mode shapes, and damping ratio were examined. Although numerous studies have addressed the static mechanical behaviour of FDM parts, there remains a significant gap in understanding how internal structural features and porosity influence their vibrational response. To address this, a total of seventy-two specimens were fabricated with varying parameter combinations, and their dynamic responses were evaluated through frequency response functions (FRFs) obtained via the impact hammer test. Damping characteristics were extracted using the peak-picking (half power) method. Additionally, the influence of internal porosity on damping behaviour was assessed by comparing the actual and theoretical masses of the specimens. The findings indicate that both natural frequencies and damping ratios are strongly influenced by the internal structure of the printed components. In particular, gyroid and cubic infill patterns increased structural stiffness and resulted in higher resonant frequencies, while low infill densities and triangle patterns contributed to enhanced damping capacity. Response Surface Methodology (RSM) was employed to develop mathematical models describing the parameter effects, providing predictive tools for applications sensitive to vibration. The high R^2^ values obtained in the RSM models based on the input variables show that these variables explain the effects of these variables on both natural frequency and damping ratio with high accuracy. The models developed (with R^2^ values up to 0.98) enable the prediction of modal behaviour, providing a valuable design tool for engineers optimizing vibration-sensitive components in fields such as aerospace, automotive, and electronics.

## 1. Introduction

Three-dimensional printing, a form of additive manufacturing (AM) technology, has undergone significant development over time. Originally developed for prototyping, it has recently been widely applied in various critical industries such as aerospace [1,2], biomedical [3,4], electromechanical systems [5], automotive [6], sensor technology [7,8,9], and biomechanics [10,11] due to its design flexibility, operational efficiency, cost-effectiveness and high degree of customization. FDM is one of the most widely used 3D printing techniques. It uses a layer-by-layer manufacturing process guided by computer-aided design (CAD) and computer-aided manufacturing (CAM) systems. Polymers are the most widely used materials in FDM due to their desirable properties such as low cost and light weight, which make them well suited for critical applications such as aircraft wings and wind turbine blades [12,13,14,15]. Among the various materials used in 3D printing, ABS remains one of the most widely used polymers in FDM-based applications. It is especially suitable for manufacturing complex geometries in industrial settings and is known for its excellent mechanical performance under static loading conditions [13].

A comprehensive review of the literature shows that a considerable number of studies have investigated the mechanical properties of FDM-fabricated structures, including compressive, flexural, and tensile strengths, as well as impact resistance and fracture toughness. Gómez-Gras et al. [16] examined the mechanical behaviour of biocompatible polycarbonate materials, emphasizing the importance of material selection for medical applications. Jayakumar et al. [17] showed that settings such as infill density, nozzle temperature, and build orientation can significantly improve the tensile, compressive, and impact strength of PLA materials. Saenz et al. [18] explored how ABS components behave under both room temperature and cryogenic conditions (77 K), highlighting the influence of temperature on mechanical performance. Lee and Wu [19] reported that carbon fibre-reinforced polymers exhibit notable improvements in both stiffness and strength. Camargo et al. [20] evaluated the mechanical performance of PLA–graphene composite filaments while focusing on the structural strength of cellulose-based tablets produced via 3D printing. Collectively, these studies underline the significant impact of printing parameters and material type on the mechanical behaviour of FDM-printed structures [21,22,23,24,25]. Additionally, the effect of critical key process parameters—such as nozzle diameter [26], layer thickness [27,28], raster angle and width [29,30,31], infill pattern [32], infill density [33], and build orientation [34,35]—have been widely studied in relation to mechanical performance. However, much of this work has focused on static properties, and the connection between internal geometry and vibrational response remains loosely defined [36].

In contrast, research focusing on the dynamic and vibrational behaviour of 3D-printed parts remains limited [37,38,39]. In particular, damping behaviour—one of the most complex and application-critical aspects of vibration response—has not been thoroughly quantified in relation to controllable FDM parameters. Several notable efforts have begun to address this gap. Sbriglia et al. [40] examined FDM operational conditions—such as layer thickness and print speed—using modal analysis techniques with an electrodynamic shaker and accelerometers. Özcanlı [41] investigated the modal and static structural characteristics of lattice-structured components produced using 3D printing. The natural frequencies and stiffness of the structures were determined experimentally, and results were validated by comparison with simulations of computer-aided Finite Element Analysis (FEA). Results show that the manufacturing parameters have a significant impact on the static and modal responses of the structures. Iyibilgin et al. [42] experimentally examined the modal characteristics of 3D-printed beams with various internal geometries. They evaluated the effects of internal geometries such as rectangular, circular, honeycomb, square, and triangular structures on damping ratios and natural frequencies. Their findings showed that the best damping performance was obtained in beams with a honeycomb internal structure, while the highest stiffness was obtained in solid specimens.

Kannan and Ramamoorthy [43] compared the modal and mechanical properties of PC, ABS, and PC-ABS components, concluding that PC-ABS had higher natural frequencies with superior load-carrying capacity. In another study, Kannan et al. [44] found that PETG samples reinforced with short carbon fibres demonstrated increased elastic modulus and tensile strength, which led to higher natural frequencies.

Rajkumar [45] analysed the influence of various infill patterns—such as triangular, cubic, and concentric—as well as different printing orientations on the mechanical properties of PLA components. Results from bending tests and modal analyses indicated a strong correlation between elastic modulus and natural frequency. Similarly, Pandya [46] investigated delaminated PLA beams, showing that increased delamination reduced natural frequencies. Nguyen et al. [36] conducted a modal analysis of 3D-printed polymer structures to investigate their vibrational characteristics. The type of bed adhesion used during printing was found to significantly affect the dynamic behaviour of the components; for instance, specimens printed with skirt adhesion exhibited higher dynamic modulus and damping ratios.

Recent research by Medel et al. [47] analysed the dynamic behaviour of 3D-printed PLA samples by Laser Doppler Vibrometry (LDV). Their research showed that the damping and stiffness properties of the materials are significantly affected by changes in printing parameters such as layer height, nozzle temperature, print speed, and raster angle. Higher nozzle temperatures and lower printing speeds resulted in improved inter-filament bonding and enhanced mechanical performance.

He et al. [26] experimentally investigated the damping behaviour of ABS cantilever beams by examining the effects of nozzle diameter, infill density, and infill pattern. Higher infill density was found to reduce the damping ratio, while nozzle diameter showed a notable effect on damping behaviour. These findings, supported by statistical and theoretical analyses, highlight the significance of printing parameters in controlling the vibration response of structures fabricated using the Material Extrusion (MEX) process. Another study [48] analysed the flexural characteristics and the effectiveness of vibration damping in panels with various infill patterns, such as Grid, Cross, and Trihexagon, as well as different infill ratios. The findings from the flexural tests revealed that the specimens with the Cross pattern demonstrated the highest level of damping capability.

Although the existing papers provide important information on the mechanical and vibrational behaviour of 3D-printed structures [49,50,51,52,53,54], some critical research gaps remain unaddressed. In particular, the lack of a direct mathematical relationship between printing parameters and damping characteristics limits the predictive capacity of existing analyses. Furthermore, in most studies the effect of porosity, which naturally arises from the FDM process, has been overlooked. In addition, inconsistencies encountered in damping measurements and the lack of standardized evaluation protocols raise questions about the repeatability and reliability of the obtained damping values.

Despite previous efforts, the damping behaviour of 3D-printed structures remains insufficiently explored. To address this gap, the present study differs from the work of He et al. [26] by comprehensively evaluating the effects of 3D printing parameters—specifically nozzle diameter, infill density, and infill pattern—while explicitly incorporating porosity as an internal feature often overlooked in previous analyses. EMA was performed using FRFs obtained from multiple measurement points, enabling detailed examination of fundamental frequencies, damping coefficients, and modal configurations characteristic of the structures. Additionally, separate predictive models were developed for each printing parameter using RSM, facilitating both visual and statistical interpretations of their influences. To the best of our knowledge, no previous study has investigated all four infill patterns with separate RSM models. This integrated approach provides a comprehensive framework for evaluating and predicting the vibrational behaviour of FDM-printed ABS beams, thus offering practical design guidance for vibration-critical applications. Understanding the relationship between FDM printing parameters and dynamic properties such as natural frequency and damping ratio is particularly critical in these application areas. Components used in aerospace, automotive, or electronic systems often operate under dynamic loading, where vibration performance directly affects functionality, durability, and noise control. Therefore, the findings of this study aim to provide a practical framework for optimizing such components during the design phase.

## 2. Materials and Methods

### 2.1. Experimental Design and 3D-Printed Specimen Fabrication

**Figure 1:**
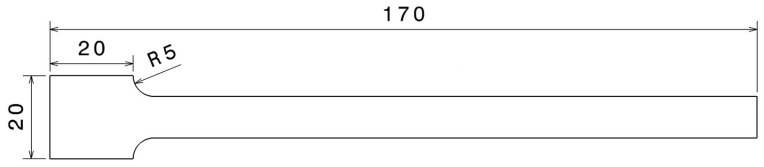
Dimensions of the 3D-printed specimens. Tested beam length: 150 mm; width: 10 mm; thickness: 3 mm. Bulk volume: 5.73 cm^3^.

The scope of this study is limited to the influence of geometrical and process parameters on vibrational characteristics, with material kept constant. ABS was chosen as a representative engineering-grade thermoplastic for 3D printing because of its remarkable attributes, such as durability, rigidity, robustness, and chemical stability [33], and widespread use in structural FDM. The specimen was designed as a cantilever beam, with the geometry shown in Figure 1, which simplifies the printing process.

**Figure 2:**
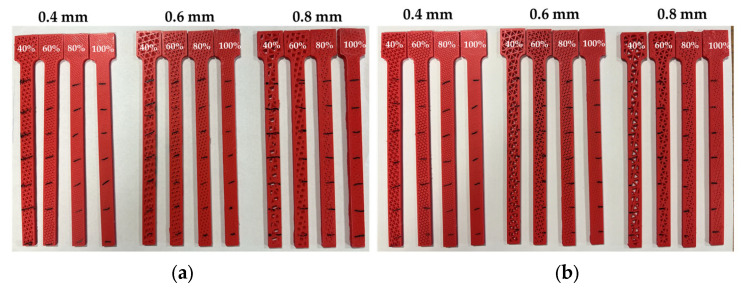

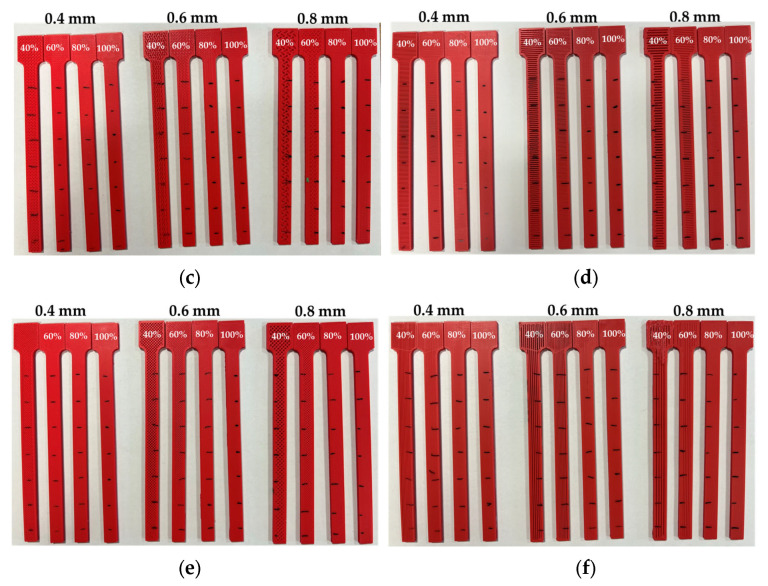
3D-printed specimens, (**a**) cubic infill pattern, (**b**) triangle infill pattern, (**c**) gyroid infill pattern, (**d**) line infill pattern with 0° raster angle, (**e**) line infill pattern with 45° raster angle, and (**f**) line infill pattern with 90° raster angle.

Initially, it is crucial to identify the parameters that are the focus of this research. Table 1 and Table 2 represent the process parameters and the mechanical properties of the ABS filament, along with the respective levels used in the experimental study. In this study, all specimens were sliced using Ultimaker Cura, where the extrusion width is automatically set equal to the nozzle diameter, and the flow rate remains at 100%. Therefore, for nozzle diameters of 0.4 mm, 0.6 mm, and 0.8 mm, the corresponding extrusion widths were 0.4 mm, 0.6 mm, and 0.8 mm, respectively. Additionally, four different infill densities (40%, 60%, 80%, and 100%) and four different infill patterns (line, gyroid, cubic, and triangle) were applied. A more detailed analysis was carried out by considering three different raster angles (0°, ±45° and 90°) especially for the samples with Line infill pattern. The raster angle refers to the orientation of the extruded filament paths within each layer, measured relative to the specimen’s longitudinal axis (X-direction). For example, a raster angle of 0° indicates filament paths aligned along the length of the beam, while ±45° and 90° correspond to diagonal and transverse orientations, respectively. A full factorial experimental design was implemented to systematically evaluate the combined and individual effects of nozzle size, infill density, infill pattern, and infill angle on dynamic characteristics. In total, 72 unique specimens (Figure 2) were designed considering all possible combinations of these parameters. The selected nozzle diameters represent common practical sizes in FDM systems, offering a range from fine-detail to high-speed printing. Infill densities were chosen to represent low, medium, and high material utilization scenarios relevant for structural and vibration-critical applications. Raster angles were chosen to explore anisotropy effects based on common industrial practices. This wide variety of parameters allows a versatile and comprehensive study of the effects on the natural frequencies and damping ratios of FDM structures.

The specimen’s CAD model was created using SolidWorks^©^ and imported into Ultimaker Cura^©^ (The Netherlands) for slicing and the configuration of process parameters. Most of the printing parameters were based on Cura’s default settings, with specific modifications aimed at achieving a balance between surface quality and production efficiency. The selected parameters included a layer height of 0.06 mm, wall thickness of 1.05 mm, nozzle travel speed of 120 mm/s, print speed of 45 mm/s, nozzle temperature of 240 °C, and bed temperature of 90 °C. Although Cura’s default high-resolution settings for Ultimaker printers typically use a 0.05 mm layer height with a 0.25 mm nozzle, the choice of 0.06 mm in this study ensured compatibility with the larger nozzle diameters (0.4 mm, 0.6 mm, and 0.8 mm) while still maintaining a high-quality surface finish. This compromise allowed for dimensional consistency across all specimens with minimal geometric deviation. To minimize interference with the internal vibrational behaviour of the printed specimens, no top solid layer was used in the printing process. The absence of a top layer helps to preserve the internal structure’s role in damping characteristics. A single bottom layer with a thickness of 0.27 mm was applied to improve bed adhesion and printing stability. These settings ensured that the vibrational properties were not masked by outer surface features, maintaining consistency across all specimens. The finalized sliced models were exported as G-CODE files and transferred to the Ultimaker 2+ printer for fabrication [26].

### 2.2. Experimental Modal Analysis Setup and Procedures

EMA was carried out to evaluate the dynamic characteristics of 3D-printed specimens under study. It is an important engineering method used to determine the dynamic properties of mechanical systems, including parameters such as natural frequencies, mode shapes, and damping ratios. This technique allows us to not only reveal dynamic behaviours that are difficult to detect by theoretical approaches but also to experimentally test the accuracy of theoretical assumptions regarding the geometry, material properties, and boundary conditions of the system [55].

**Figure 3:**
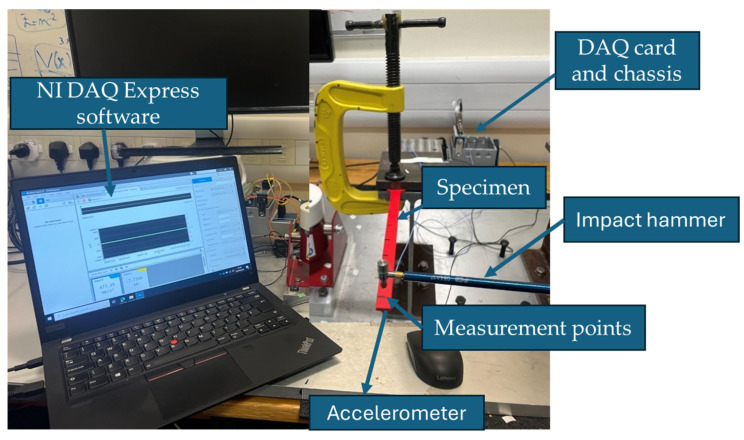
Experimental modal analysis setup.

The specimens were clamped to the testing platform with a fully constrained boundary condition applied at the fixed end (Figure 3). The clamping conditions and impact hammer test setup were kept constant and carefully controlled throughout all tests. A miniature acceleration transducer with a sensitivity of 10 mV/g (PCB 352A21 model) was attached to the free end of the beam to measure acceleration data in real time. Seven measurement points were designated along the beam, with a spacing of approximately 2 cm between consecutive points. During the test, to ensure the reliability of the damping and other dynamic response results, each specimen was excited three times using a modal impact hammer with a sensitivity of 22.5 mV/g (PCB 086E80) at a consistent excitation point. The resulting FRFs were collected and evaluated for repeatability. Averaged values were used in the analysis to reduce the influence of random variability and ensure robust comparisons. The input data from hammer and response data from accelerometer are acquired with the help of the data acquisition (DAQ) card (NI 9234) and DAQ chassis (NI 9174) (National Instruments, London, UK). The data was recorded using NI DAQ Express v5.1 software and subsequently imported into MATLAB R2022a for the extraction of FRF curves.

### 2.3. Experimental Data Process

FRF is the critical data necessary for conducting experimental modal analysis. Collected time domain data is processed to determine the FRF by converting it into the frequency domain using a fast Fourier transform. The FRF is defined as the ratio of the output response of a structure to the input force in the frequency domain. For illustration, we will examine a single-degree-of-freedom mass–spring–damper system. The equation of motion for a single-degree-of-freedom (SDOF) viscously damped mass–spring system is given by the following:

(1) $$m\ddot{x}+c\dot{x}+kx=f(t)$$

In the undamped case, the system’s natural frequency and damping ratio are as follows:

(2) $$\omega_{n}=\sqrt{\frac{k}{m}}$$

(3) $$\zeta_{r}=\frac{c}{2\sqrt{km}}$$

If the applied force is assumed to be of the form $f\left(t\right)=Fe^{i\omega t}$, then the solution takes the form $x\left(t\right)=Xe^{i\omega t}$. In this case, the system’s FRF in the receptance form can be expressed as given in Equations (4) and (5) [56].

(4) $$H\left(\omega \right)=\alpha \left(\omega \right)=\frac{1}{\left(k-\omega^{2}m\right)+i(\omega c)}$$

(5) $$\left|H\left(\omega \right)\right|=\frac{\left|X(\omega )\right|}{\left|F(\omega )\right|}=\frac{1}{\sqrt{{\left(k-\omega^{2}m\right)}^{2}+{\left(\omega c\right)}^{2}}}$$

Here, X(ω) and F(ω) represent the Fourier transforms of the displacement and force functions, respectively. In a system with *N* degrees of freedom, an element of the receptance matrix is given by Equation (6), which describes the dynamic response of an N-degree-of-freedom system in terms of its modal parameters.

(6) $$H_{ij}\left(\omega \right)=\frac{X_{i}(\omega )}{F_{j}(\omega )}=\sum_{r=1}^{N}\frac{\phi_{i}^{r}\phi_{j}^{r}/k_{r}}{{\left(1-\omega /\omega_{r}\right)}^{2}+2i\zeta_{r}(\omega /\omega_{r})}$$

where $H_{ij}\left(\omega \right)$ is the receptance function, $\phi_{i}^{r}$ and $\phi_{j}^{r}$ are the mode shape components at coordinates *i* and *j*, ω is the excitation frequency, and $\omega_{r}$ and $k_{r}$ are, respectively, the natural frequency and modal stiffness of the *r*th mode.

Various methods are available for determining damping ratios from the FRF, including the peak-picking, Circle Fit, Inverse FRF, and Least-Squares Method [55,56,57]. In this study, the peak-picking method was chosen due to its simplicity of application, the lightly damped nature of the examined systems, and the sufficient separation of their modes.

**Figure 4:**
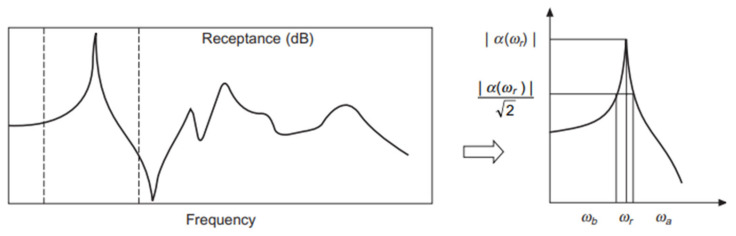
Peak-picking method [57]. The range between the dotted lines shows the peak for fundamental mode.

The peak-picking method, commonly used in experimental modal analysis, is also referred to as the half-power method. According to this approach, the natural frequency corresponding to the *r*th peak of the FRF curve ($\omega_{r}=\omega_{peak}$) is first identified. Next, the points where the signal energy drops to half (i.e., where the FRF amplitude equals $1/\sqrt{2}$ of the peak value) are determined. These points, known as half-power points, are denoted as $\omega_{a}\;$ and $\omega_{b}$, as shown in Figure 4. The damping loss factor ($\eta_{r}$) and damping ratio ($\zeta_{r}$) are then calculated using the following equations.

(7) $$\eta_{r}=\frac{\omega_{b}^{2}-\omega_{a}^{2}}{2\omega_{r}^{2}}\approx \frac{\omega_{b}-\omega_{a}}{\omega_{r}}$$

(8) $$\zeta_{r}=\frac{\omega_{b}^{2}-\omega_{a}^{2}}{4\omega_{r}^{2}}\approx \frac{\omega_{b}-\omega_{a}}{2\omega_{r}}$$

### 2.4. Response Surface Method (RSM)

An RSM model is an approximate mathematical representation of a system’s specific characteristics, providing a functional relationship between input and output parameters. Let $x_{i}\;(for\;i=1,\;2,\;\ldots ,\;n)$ be the input parameters and y the output parameter; the RSM model is represented by the following function:

(9) $$y=f(x_{1},\;x_{2},\;x_{3},\;\ldots ,\;x_{n})+\varepsilon$$

Here, ε is a statistically random error term, normally distributed with a mean of zero. For most engineering problems, a first- or second-order polynomial model is sufficient, and such a model can be expressed as follows:

(10) $$y=f(x_{1},\;x_{2},\;\ldots ,\;x_{n})+\varepsilon =\beta_{0}+\Sigma (\beta_{i}x_{i})+\Sigma (\beta_{ij}x_{i}x_{j})+\varepsilon$$

In this Equation (10), β represents the partial regression coefficients. The terms $x_{i}$ indicate the main effects of the input parameters, while the interaction terms $x_{i}x_{j}$ reflect the interaction effects between parameters i and j.

To evaluate the quality of an RSM model—specifically, how well the experimental data fits the model—the most used indicators are the coefficients R^2^, $R_{adj}^{2}$, and $R_{pred}^{2}$. For a good model, these coefficients should be close to 1. The R^2^ coefficient, which measures the correlation between the actual observed values y and the predicted values ŷ, is calculated as follows:

(11) $$R^{2}=SSR/SST$$

where SST is the total sum of squares and SSR is the regression sum of squares.

However, because adding more independent variables to the model can always increase the R^2^ value, it is not a sufficient indicator by itself. The $R_{pred}^{2}$ coefficient represents the model’s predictive power. For a reliable model, all three coefficients should be close to 1, and the difference between $R_{adj}^{2}$ and $R_{pred}^{2}$ should be less than 0.2. Given p as the number of parameters and (n−p) as the degrees of freedom, $R_{adj}^{2}$ and $R_{pred}^{2}$ are calculated as follows:

(12) $$R_{adj}^{2}=1-((SSE/(n-p))/(SST/(n-1)))\;\;\;\;\;\;\;\;=1-((n-1)/(n-p))\;*\;(1-R^{2})$$

(13) $$R_{pred}^{2}=1-PRESS/SST$$

where PRESS (Prediction Error Sum of Squares) is a measure of how well the model fits the data across the design space, representing the sum of the squares of the prediction errors [58].

## 3. Results and Discussion

**Figure 5:**
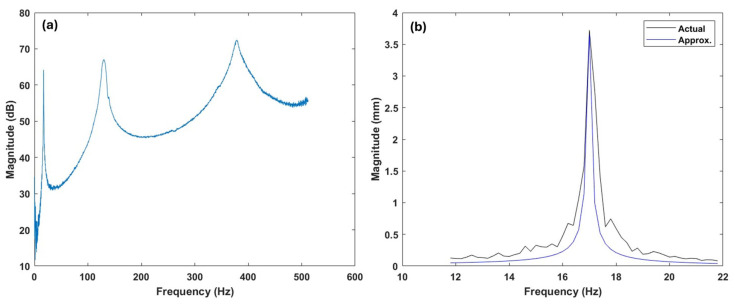
(**a**) A sample FRF for a specimen with a nozzle size of 0.6 mm, gyroid infill pattern, and 40% infill density. (**b**) Modal curve-fitted FRF for the first FRF peak.

The natural frequencies and damping ratios of the specimens were determined by EMA. Figure 5 depicts the parameter extraction process through FRF analysis. Figure 5a presents the complete FRF curve from a representative sample (gyroid infill pattern with 40% infill density and 0.6 mm nozzle size), illustrating multiple resonance peaks that correspond to the natural frequencies of the system. Figure 5b shows the initial resonance peak, where an approximate fitted curve obtained using the peak-picking method is overlaid on the actual experimental data. This curve fitting method enables accurate estimation of the natural frequency and the associated damping ratio through analysis of the resonance peak’s shape and width.

The findings of this study were assessed comparatively under two primary areas. The impact of various infill patterns on the dynamic characteristics of structural elements was examined. In this context, specific focus was placed on the line infill pattern, examining various raster angle configurations to assess natural frequencies and damping ratios. In the second part of the research, the effect of porosity on damping behaviour was comprehensively analysed by considering the parameters of infill density, infill pattern, and porosity together. The results obtained demonstrate that the manufacturing parameters applied with the infill configurations have a substantial influence on the vibrational behaviour of the materials produced by 3D printing.

### 3.1. Influence of the Infill Pattern

**Figure 6:**
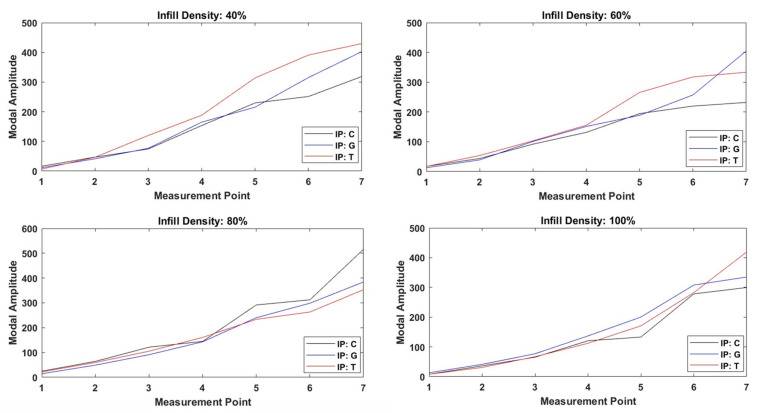
Visualization of first mode shapes for various infill patterns (IPs) and densities at constant nozzle size (0.8 mm). (IP: Infill pattern). (C: cubic, G: gyroid, T: triangle).

This work comprehensively examined the impacts of various infill patterns (cubic, triangular, gyroid, and linear), nozzle sizes, and infill densities on the natural frequencies and mode shapes of 3D-printed structures. During the experimental design process, RSM was applied individually for each infill pattern to develop mathematical models for damping ratios and natural frequencies. The results show that the infill pattern parameters have a significant effect on the dynamic response of structural members. The effects of various infill patterns on natural frequencies and damping ratios are analysed in detail, and the findings are supported by visual comparisons of the mode shapes presented in Figure 6, which illustrate deformation behaviour under resonance conditions.

The data presented in Table 3 offers a comparative evaluation of the first natural frequency and damping ratio values obtained for different infill patterns (cubic, triangle, and gyroid). When examining infill patterns at the same infill density, notable differences in natural frequency emerge. For instance, with a nozzle size of 0.8 mm and an infill density level of 100%, the gyroid pattern records the highest natural frequency of 23.607 Hz, followed by the cubic pattern at 22.793 Hz and the triangle pattern at 22.192 Hz. On the other hand, the cubic infill pattern shows the highest natural frequency at 40% infill density across all nozzle sizes. This suggests that the cubic structure performs better in terms of stiffness at lower infill levels. However, at higher infill densities, the gyroid pattern appears to offer a more robust and integrated internal architecture, reflected in its overall higher natural frequencies. The gyroid pattern exhibits interconnected curved channels that distribute stresses uniformly, thereby enhancing structural stiffness and increasing natural frequencies [59]. Regarding the damping ratio values, the maximum and minimum values (0.0153 and 0.0057, respectively) were observed in specimens with the cubic infill pattern. Triangle-patterned specimens are characterized by a moderate frequency range and relatively high damping ratios. In many cases, the damping values are around 0.01, indicating that this pattern may be effective in vibration damping. This implies that the triangle pattern offers enhanced energy absorption, although it may result in a slight reduction in stiffness. Triangular patterns create defined internal friction zones, leading to improved energy dissipation and thus higher damping ratios [60].

In Figure 6, the variation in mode shape with respect to the measurement points is analysed for each infill density level. It is observed that different infill patterns produce noticeable differences between the mode shape profiles, affecting the vibration behaviour of the internal structure. Notably, even under the 100% infill condition, differences in modal amplitude were observed across samples with different infill patterns. This is attributed to the fact that slicing software (Ultimaker Cura v5.9) retains the geometry-specific toolpath of each pattern at full density, resulting in residual voids of varying size, orientation, and distribution. These subtle structural distinctions affect local stiffness, bonding uniformity, and vibrational energy dissipation mechanisms. As a result, the infill pattern continues to influence the modal response, including amplitude, even when the nominal density is held constant.

As the density of the structure increases, the mode shapes become smoother and more like the theoretical first mode shape of cantilever beam. This behaviour can be explained by the increased internal compactness and uniformity associated with higher infill densities, which help distribute stress more evenly across the structure. As supported by previous studies, such uniform material distribution enhances both stiffness and tensile strength by reducing localized deformation zones and minimizing internal irregularities [61,62,63]. On the other hand, lower infill densities introduce higher porosity and irregular stress transmission paths, which can cause variations in local stiffness and lead to deviations in the mode shape. This explains why the higher infill specimens display the most uniform and stable deformation patterns observed in the figure.

#### 3.1.1. Cubic Infill Pattern

**Figure 7:**
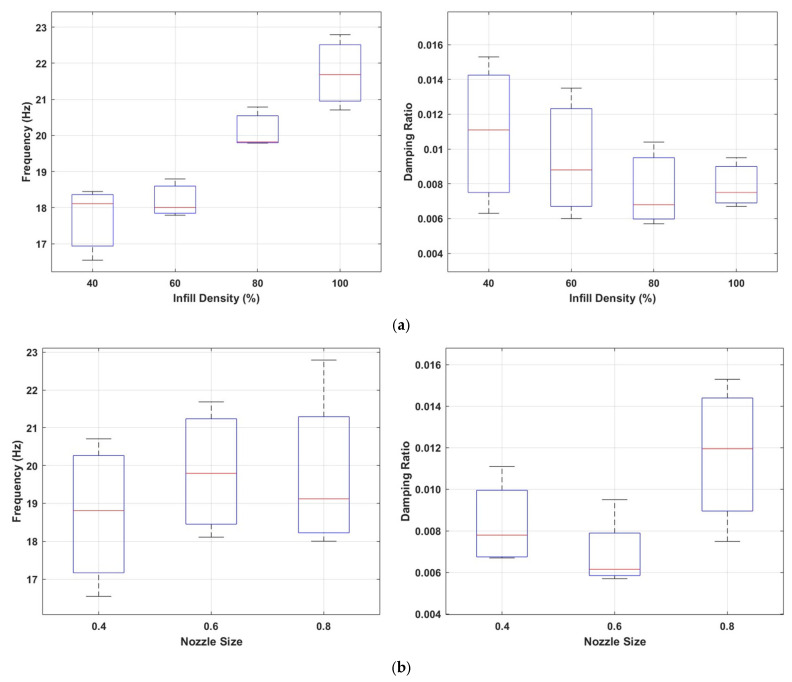
Comparison of natural frequencies and damping ratios for the cubic infill pattern (**a**) at different infill densities; (**b**) with varying nozzle sizes.

Figure 7a shows the effects of different infill densities (40%, 60%, 80%, and 100%) on the first natural frequency (Hz) and damping ratio. The data shows that the natural frequency increases significantly as the infill density increases. This trend indicates that the structural rigidity increases with the increase in the internal infill and, accordingly, an increase in the resonance frequency of the system occurs. It is observed that the frequency distribution becomes narrower and more stable, especially at infill densities of 60% and 80%. This shows that these infill ratios make the internal structure more homogeneous and structurally balanced.

When evaluated in terms of damping ratios, a significant decrease in damping ratios is observed with the increase in infill density. At low infill densities (especially 40% and 60%), the wider distribution of damping ratios indicates the formation of a more irregular and more effective interior structure in terms of energy absorption. On the other hand, at high infill densities (80% and 100%), energy dissipation decreases due to the increased stiffness of the structure, resulting in a more rigid structural behaviour that transmits vibrations more efficiently.

Specimens manufactured with a 0.6 mm nozzle typically exhibited higher natural frequency values than those produced with a 0.4 mm nozzle, even when the infill density was kept constant (as shown in Figure 7b). This can be explained by the fact that the thicker extruded filament lines create a stiffer internal grid structure, thus increasing the overall structural rigidity. As the nozzle diameter increased, an increase in the damping ratio was not observed. However, this trend was not linear when it came to damping behaviour. The damping ratio at 0.6 mm nozzle diameter was lower than both 0.4 mm and 0.8 mm configurations. Notably, specimens printed with a 0.8 mm nozzle exhibited a substantial increase in damping ratio. This suggests that larger nozzle diameters may introduce more internal friction and bonding irregularities, resulting in greater energy dissipation. These findings indicate that nozzle diameter has a non-linear and pattern-dependent influence on the dynamic response of 3D-printed components, affecting both stiffness and damping capacity in complex ways.

**Figure 8:**
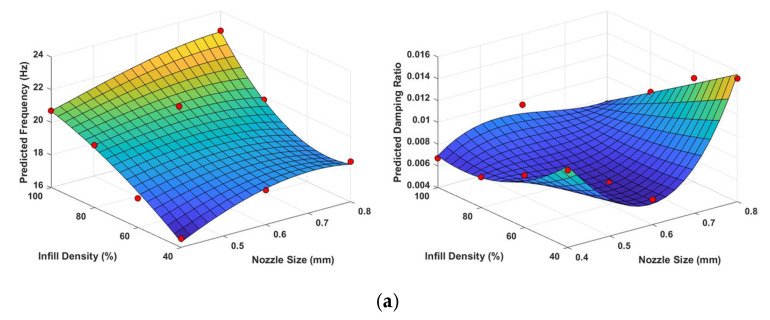

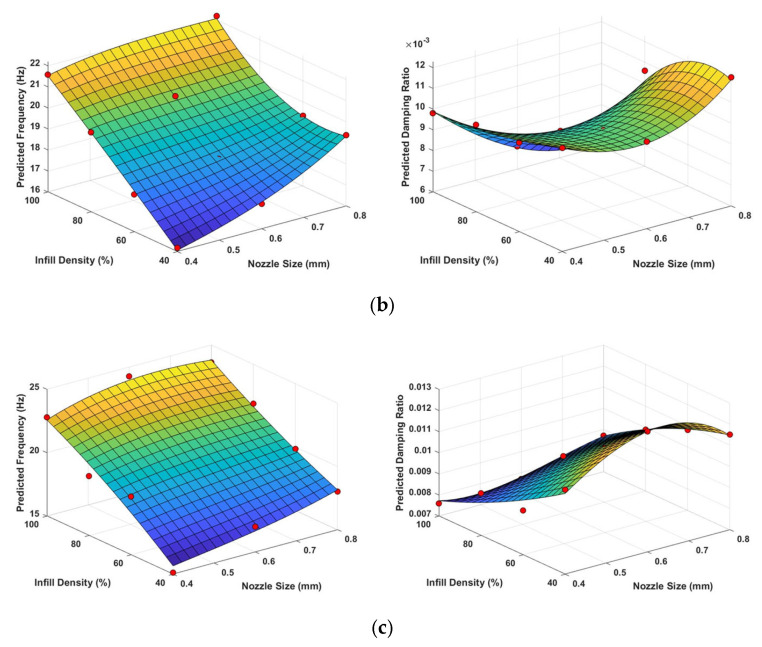
RSM plots of different infill patterns as functions of infill density and nozzle size. (**a**) Cubic; (**b**) triangle; (**c**) gyroid. Red dots represent the raw experimental data.

Equations (14) and (15) represent the third-order polynomial predictive models for natural frequency and damping ratio, respectively, for the cubic pattern, based on the results obtained from the RSM (Figure 8a). Moreover, Table 4 presents the statistical values for each infill pattern. As shown in Table 4, the R^2^ and $R_{adj}^{2}$ values of C_1_ represent the quadratic predictive model of the cubic pattern, and the estimated natural frequency models are above 0.85, indicating a good fit. However, the relatively low estimated $R_{pred}^{2}$ values reveal that the models have limited predictive capacity. On the other hand, the general *R* values of the models developed for the damping ratio are at lower levels compared to the models for natural frequencies. The low $R_{pred}^{2}$ values especially show that the accuracy level of the damping ratio models is limited and therefore their predictive performance is less reliable than the natural frequency models.

(14) $$y_{f_{C}}=1.1292x_{1}+0.5533x_{2}+0.6467x_{1}^{2}-0.1123x_{1}x_{2}-0.00288x_{2}^{2}\;\;\;\;\;-0.05389x_{1}^{3}+{0.00245x}_{1}^{2}x_{2}+0.000595x_{1}x_{2}^{2}$$

(15) $$y_{d_{C}}=0.0312x_{1}-0.0018x_{2}-0.0092x_{1}^{2}+0.0005x_{1}x_{2}+\left(4.177e-6\right)x_{2}^{2}\;\;\;\;\;\;\;-0.00069x_{1}^{3}-{\left(3.768e-5\right)x}_{1}^{2}x_{2}-(5.156e-7)x_{1}x_{2}^{2}$$

where $x_{1}\;$ and $x_{2}$ represent the input parameters NS and ID, respectively. $y_{f_{C}}$ denotes the predicted response function for natural frequency while $y_{d_{C}}$ represents the predicted response function for damping ratio.

In the literature, second-order models are commonly used in RSM applications. However, in this study, third-order polynomial expansions (‘*poly32*’ command used in MATLAB for all infill patterns) were also considered to improve the $R^{2}$, $R_{adj}^{2}$, and especially $R_{pred}^{2}$ values. It was observed that better results were obtained. As shown in Table 4, the $R^{2}$ and $R_{adj}^{2}$ values of C_2_—representing the third-order polynomial predictive model (with a polynomial degree of 3 for NS and 2 for ID) for the cubic infill pattern—are higher than those of C_1_, indicating an improved model fit. The statistical significance of the RSM models (Figure 8) was confirmed by ANOVA (Appendix A Table A1), where both linear and nonlinear effects were analysed separately. As indicated by the results, the cubic pattern’s natural frequency model is predominantly governed by linear effects, while the damping ratio exhibits notable nonlinear contributions. This reflects a more complex internal vibration energy dissipation behaviour despite relatively predictable stiffness trends.

#### 3.1.2. Triangle Infill Pattern

**Figure 9:**
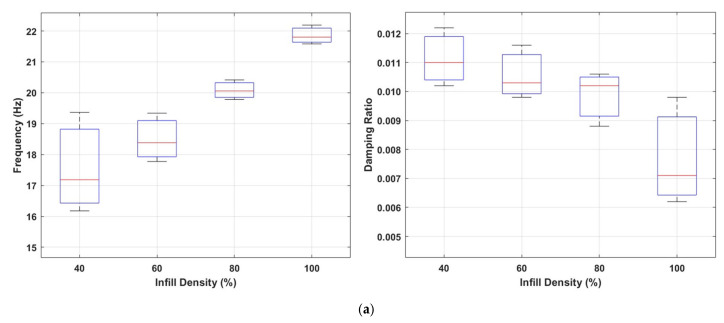

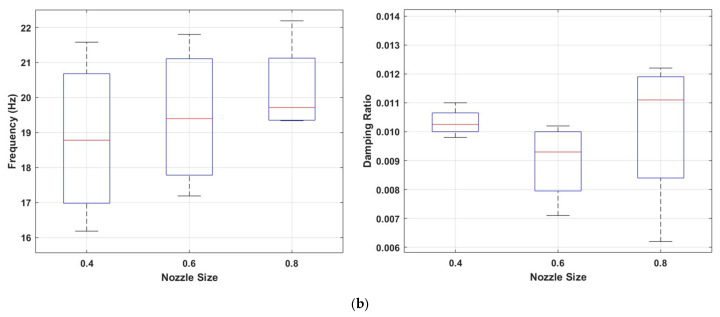
Comparison of natural frequencies and damping ratios for the triangle infill pattern (**a**) at different infill densities; (**b**) with varying nozzle sizes.

Figure 9 illustrates the effects of infill density and nozzle diameter parameters on the first natural frequency and damping ratio. Similar to the cubic infill pattern, the natural frequency increases substantially as the infill density increases, while the damping ratio decreases, as illustrated in Figure 9a. This indicates that higher infill levels enhance the structural stiffness, thereby increasing the resonance frequency, but at the same time reduce the structure’s energy dissipation capacity. Notably, at 100% infill density, the frequency reaches its highest level, whereas the damping ratio drops to its lowest.

Figure 9b shows the influence of nozzle diameter on the dynamic properties. As the nozzle diameter increases, the natural frequency generally rises, which can be attributed to the increased structural rigidity provided by thicker extrusion paths. However, when examined in terms of damping ratio, there is a different trend. At low infill densities, the damping ratio distribution is more limited. However, as the infill density increases, this situation reverses; at high infill levels, the triangle pattern exhibits a wider distribution. Higher damping ratios were obtained, especially in samples produced with 0.8 mm nozzle diameter. Triangular infill patterns tend to introduce more frequent intersections and angular joints, which can act as stress concentrators and promote localized energy dissipation through viscoelastic and frictional effects [32]. Moreover, the discontinuity in load paths and the high rotational symmetry of these structures are believed to enhance internal micro-motion, thereby increasing damping capacity [61].

**Figure 10:**
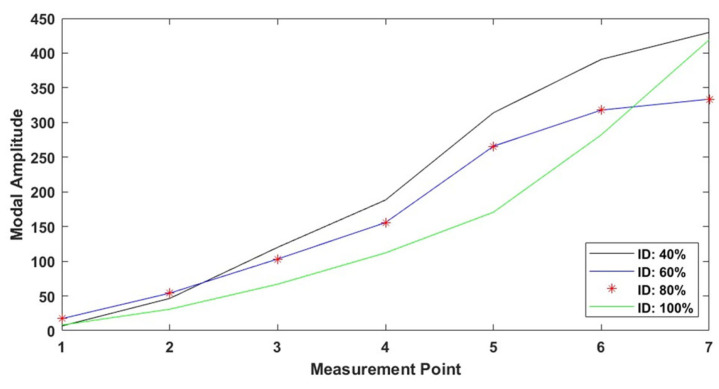
Mode shape variation for triangle infill pattern at varying infill densities (IDs) (0.8 mm nozzle size).

Figure 10 shows the effect of different infill densities on the first mode shape of the specimens produced using a nozzle diameter of 0.8 mm and a triangular infill pattern. The graph shows the modal amplitude change according to the measurement points. It can be seen that the modal amplitude values generally decrease (except for the free end) as the infill density increases. This indicates that higher infill rates limit the vibration amplitude by increasing the stiffness of the structure. When infill density is 100%, the mode shape becomes very smooth and more similar to the theoretical mode shape. In other words, as the density increases, there is a change implying that the homogeneous distribution in the material is more, which increases the continuity of the modal amplitudes.

Equations (16) and (17) illustrate the predictive models (third-order polynomial) for natural frequency and damping ratio, respectively, for the triangle pattern, derived from the findings of the RSM (Figure 8b). Table 5 presents the statistical performance metrics of two different RSMs (T_1_ and T_2_) developed for predicting natural frequencies and damping ratios. The T_1_ model represents a second-order (quadratic) polynomial, while the T_2_ model is based on a third-order polynomial.

In terms of natural frequencies, the T_2_ model shows higher $R^{2}$ (0.992) and $R_{adj}^{2}$ (0.978) values compared to the T_1_ model, indicating a better overall fit to the experimental data. Although the predicted $R_{pred}^{2}$ value of T_2_ (0.851) is slightly lower than that of T_1_ (0.860), the improved *p*-value (0.00052) and higher F-value (69.5) demonstrate that T_2_ is statistically more robust and significant. A similar trend is observed for damping ratios. The T_2_ model outperforms T_1_ with higher $R^{2}$ (0.984) and $R_{adj}^{2}$ (0.956) values. In addition, T_2_ model presents a lower *p*-value (0.00195) and a significantly higher F-value (35.3), indicating greater statistical significance. Most importantly, the value 0.738, which indicates the predictive performance of the T_2_ model, is significantly higher than the value of the T_1_ model (0.468), suggesting an improved predictive capability.

(16) $$y_{f_{T}}=4.7936x_{1}+0.2311x_{2}-0.6286x_{1}^{2}-0.02135x_{1}x_{2}-0.001302x_{2}^{2}\;\;\;\;+0.04675x_{1}^{3}-{0.00273x}_{1}^{2}x_{2}+0.000306x_{1}x_{2}^{2}$$

(17) $$\begin{array}{ll} y_{d_{T}}=0.01092x_{1} & -0.000382x_{2}-0.002592x_{1}^{2}+\left(8.6313e-5\right)x_{1}x_{2}\\ & +\left(2.8438e-6\right)x_{2}^{2}+0.000166x_{1}^{3}-{\left(1.3125e-6\right)x}_{1}^{2}x_{2}\\ & -(6.4063e-7)x_{1}x_{2}^{2} \end{array}$$

The more statistical analysis results (Table A1) confirm that both natural frequency and damping ratio are strongly influenced by linear and nonlinear terms in the triangle pattern. This highlights the triangle structure’s sensitivity to parameter variations, especially for damping behaviour.

#### 3.1.3. Gyroid Infill Pattern

**Figure 11:**
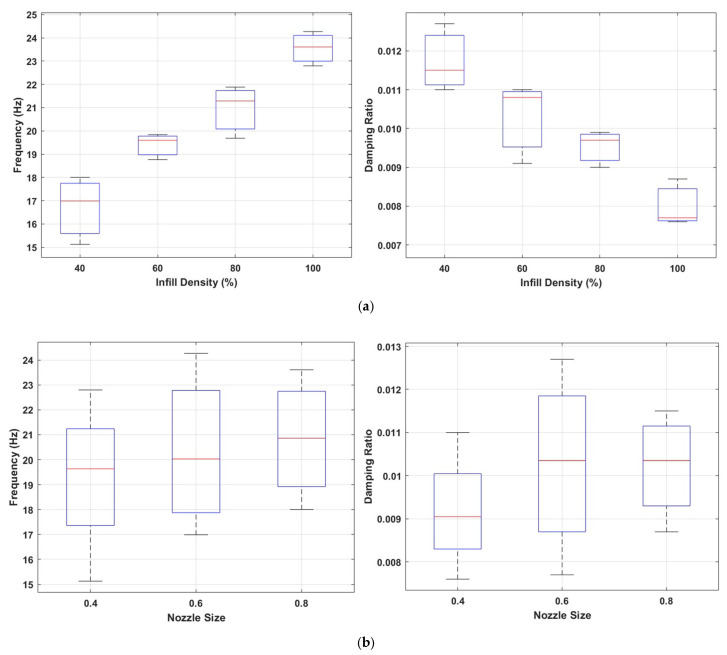
Comparison of natural frequencies and damping ratios for the gyroid infill pattern (**a**) at different infill densities; (**b**) with varying nozzle sizes.

The influence of infill density and nozzle diameter on the natural frequency and damping ratio of structures with a gyroid infill pattern is shown in Figure 11. Figure 11a illustrates an increase in infill density leads to enhanced structural stiffness, resulting in a significant rise in natural frequency. The gyroid structure forms a continuous and periodic surface with minimal sharp transitions. This geometry enables efficient stress distribution and increases global stiffness, as reflected by higher natural frequencies. However, the smooth connectivity reduces localized strain gradients and internal friction, which reduces its damping. Figure 11b demonstrates that nozzle diameter also affects the dynamic response; larger nozzle diameters tend to increase both the natural frequency and the damping ratio.

**Figure 12:**
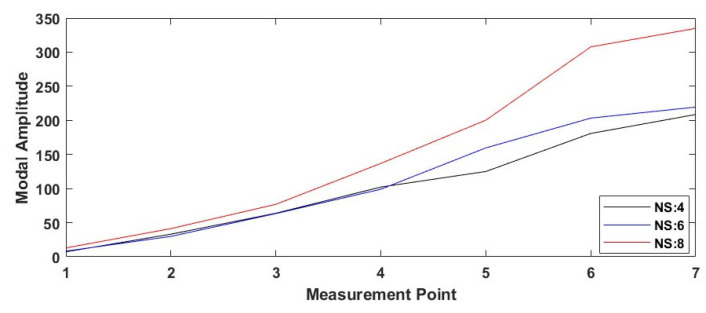
Mode shape variation for gyroid infill pattern under varying nozzle sizes (NSs).

Figure 12 shows the effect of different nozzle sizes on the mode shape for specimens with the gyroid infill pattern. The data shows that the modal amplitudes increase as the nozzle diameter increases. In particular, the samples with a nozzle diameter of 0.8 mm exhibited the highest modal amplitude values at all measurement points. This reveals that thicker extruded filaments can significantly affect the vibration amplitude as well as increasing the structural stiffness.

Equations (18) and (19) represent predictive model of natural frequency and damping ratio of gyroid infill pattern. The statistical performance of two predictive models, G_1_ (quadratic) and G_2_ (third order polynomial), developed to estimate the damping ratios and natural frequencies of structures with a gyroid infill pattern, is outlined in Table 6 and Figure 8c. Both models exhibit strong goodness-of-fit, as indicated by their $R^{2}$ and $R_{adj}^{2}$ values. However, G_1_ demonstrates superior predictive capability for natural frequencies, with a higher predicted $R_{pred}^{2}$ value of 0.832, compared to 0.263 for G_2_. In contrast, G_2_ outperforms G_1_ in predicting damping ratios, exhibiting higher $R^{2}$, $R_{adj}^{2}$, and $R_{pred}^{2}$ values. Additionally, G_2_ shows greater statistical significance in modelling damping behaviour, as evidenced by its higher F-value of 26.8. According to the ANOVA results provided in Appendix A, natural frequency variations in the gyroid pattern are primarily governed by linear effects, indicating a predictable stiffness response. In contrast, damping behaviour shows moderate nonlinear influence, likely due to the complex infill geometry within the structure.

(18) $$y_{f_{T}}=10.685+0.87703x_{1}+0.65148x_{2}-0.011264x_{1}x_{2}+0.219696x_{1}^{2}+0.000533x_{2}^{2}$$

(19) $$y_{d_{T}}=000432x_{1}+\left(8.1e-5\right)x_{2}-\left(6.8437e-5\right)x_{1}^{2}-\left(7.4375e-5\right)x_{1}x_{2}+\left(8.75e-7\right)x_{2}^{2}\;\;\;\;-(3.5781e-5)x_{1}^{3}-{\left(8.125e-6\right)x}_{1}^{2}x_{2}-(1.5625e-7)x_{1}x_{2}^{2}$$

#### 3.1.4. Line Infill Patterns

**Figure 13:**
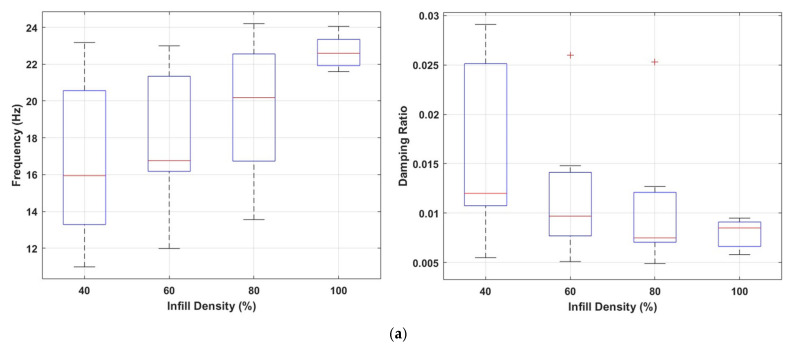

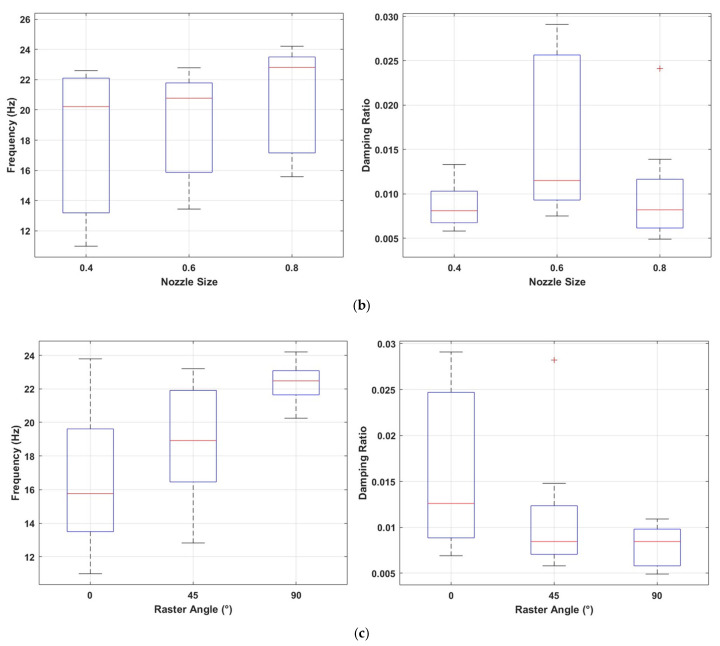
Comparison of natural frequencies and damping ratios for the line infill pattern (**a**) under varying infill densities; (**b**) with different nozzle sizes; (**c**) at different infill angles.

The effects of infill density, raster angle, and nozzle size on the natural frequency and damping ratio of 3D-printed samples produced with the line infill pattern are detailed in Figure 13. It is indicated that an increase in infill density leads to a substantial increase in natural frequency, while the damping ratio usually declines (Figure 13a). Moreover, both the raster angle and nozzle size significantly affect the results. Samples with a 90° raster angle and larger nozzle sizes (Figure 13b,c) typically show higher frequencies due to a more robust internal support structure. In contrast, the damping behaviour is more pronounced with variations in raster angle, particularly at lower densities, where 0° configurations are more effective in energy absorption. Raster angle significantly influences structural anisotropy due to directional filament alignment [60]. A raster angle parallel to the loading direction typically increases stiffness and thus natural frequency, whereas perpendicular or angled raster directions may enhance damping by increasing internal frictional interfaces.

As the nozzle size increases, the strength and therefore natural frequency values also increase [64], with the highest frequencies observed at 0.8 mm. In terms of damping ratio, the 0.6 mm nozzle shows higher and more variable values compared to the others. This indicates that a medium-sized nozzle provides better vibration damping, while a larger nozzle results in greater structural stiffness. Increasing nozzle diameter generally results in thicker extruded filaments, thus affecting the microstructure by altering interlayer bonding characteristics. Larger nozzles may improve bonding consistency, potentially affecting both stiffness and damping through changes in the structural integrity [65].

**Figure 14:**
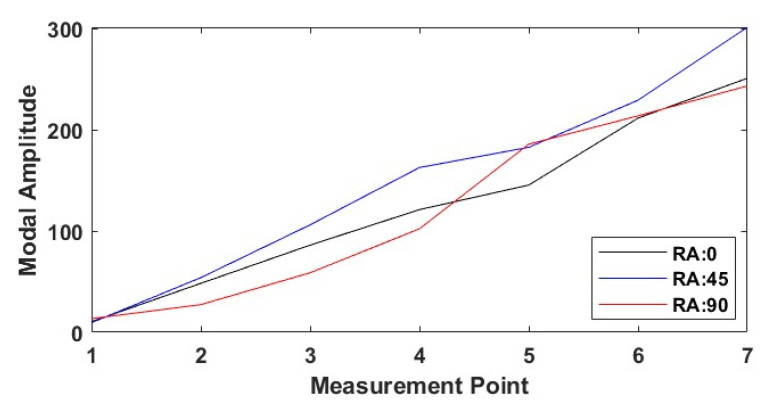
Mode shape comparison for different raster angles in line infill pattern (NS: 6 mm, RA: raster angle).

Figure 14 shows how the modal amplitudes vary with respect to the measurement points for different raster angles (0°, 45°, 90°) for specimens with the line infill pattern and a nozzle diameter of 0.6 mm. According to the data, the 45° raster angle exhibits the highest modal amplitudes in general, indicating a more flexible vibration behaviour in this direction. The 0° raster angle is intermediate, while the specimens produced at a 90° raster angle show lower modal amplitudes. This shows that the raster angle influences the internal structure orientation and stiffness and thus can directly affect the vibration response.

For the line infill pattern, the RSM yielded reliable models for both prediction of natural frequency and damping ratio predictions (Table 7). The model for natural frequency shows good fit with an $R^{2}$ of 0.892 and $R_{pred}^{2}$ of 0.801, indicating acceptable predictive performance. The model for damping ratio is slightly less accurate, with an $R^{2}$ value of 0.772 and $R_{pred}^{2}\;$ value of 0.565. The dynamic properties of the 3D-printed specimens are affected by nozzle size $\left(x_{1}\right),$ infill angle $\;(x_{2}$), and infill density ${(x}_{3})$, as represented in Equations (20) and (21), which feature quadratic, linear, interaction terms.

According to the ANOVA results (Appendix A), both linear and nonlinear components significantly affect the variation in natural frequency and damping ratio for the line pattern. This highlights the importance of inter-parameter interactions and the role of anisotropic filament orientation in governing the vibrational response.

(20) $$y_{f_{G}}=3.475+0.5791x_{1}+0.1516x_{2}+0.07276x_{2}-0.00432x_{1}x_{2}-0.00943x_{1}x_{3}\;\;\;\;-0.00134x_{2}x_{3}+0.0767x_{1}^{2}+0.000362x_{2}^{2}+0.00103x_{3}^{2}$$

(21) $$\begin{array}{ll} y_{d_{G}}=-0.0258 & +0.0214x_{1}-0.000129x_{2}-0.000439x_{2}-\left(2.333e-5\right)x_{1}x_{2}\\ & +\left(2.2917e-6\right)x_{1}x_{3}+\left(2.1093e-6\right)x_{2}x_{3}-0.00169x_{1}^{2}\\ & +\left(4.301e-7\right)x_{2}^{2}+\left(1.431e-6\right)x_{3}^{2} \end{array}$$

### 3.2. Influence of Porosity

Porosity represents an essential structural characteristic of 3D-printed components, significantly influenced by infill rates and manufacturing parameters. It is generally determined by contrasting the theoretical (ideal) mass or volume of the component with its actual (measured) mass or volume. In this research, porosity was assessed through mass measurements (Equation (22)) [66], wherein the actual mass of each printed specimen was compared to the theoretical mass obtained from the CAD model and the known density of the material.

(22) $$Porosity\;\left(\%\right)=\left(1-\frac{m_{actual}}{m_{theoretical}}\right)\times 100$$

$m_{actual}$: The actual measured mass of the printed part.
$m_{theortical}$: The mass the part would have if it were printed solid (100% infill).

**Figure 15:**
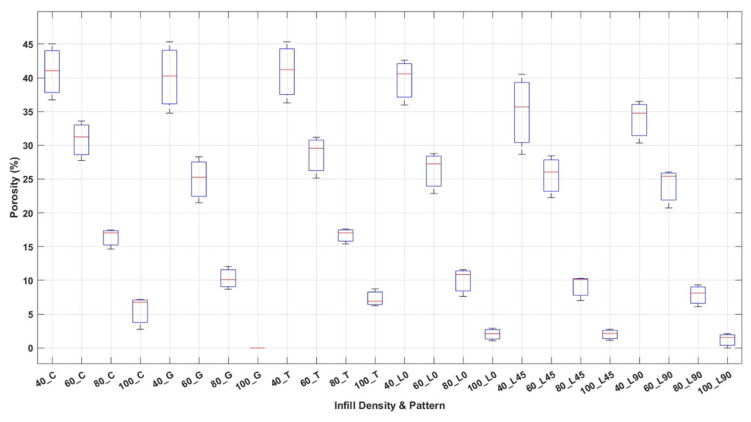
Porosity distribution for various infill patterns and densities in 3D-printed specimens.

The porosity values associated with different infill patterns—cubic, gyroid, triangle, and line—at multiple infill angles and densities are illustrated in Figure 15. The data was visualized using boxplots to capture variability and outliers across each group. A distinct inverse correlation can be identified between infill density and porosity. With an increase in infill density, there is a marked reduction in porosity, suggesting that higher infill levels diminish the volume of internal voids within the printed structures. This trend is evident across all categories of infill patterns. In addition, significant disparities are noted among different infill designs. At lower densities, cubic, triangle, and gyroid patterns all show high porosity levels, whereas the line pattern, especially at a 90° orientation, reveals significantly reduced porosity, indicating a denser and more compact internal configuration. In the gyroid pattern, the porosity distribution was more homogeneous, and the most compact structure was obtained with almost zero porosity at 100% infill density. In cubic and line patterns, on the other hand, a considerable amount of porosity is observed at the highest infill density. Especially in the cubic pattern, pore formation is higher compared to other patterns. These findings underscore the substantial impact of infill pattern and orientation on the mechanical characteristics, internal void structure, and overall manufacturing quality of 3D-printed parts.

Although the infill density was set to 100%, the slicing software (Ultimaker Cura) does not generate a truly solid internal structure. Each pattern retains its distinct toolpath geometry, and the infill line distance is automatically adjusted based on the selected pattern. As a result, small internal voids remain, and their distribution varies with pattern type. This leads to minor differences in the internal architecture even at nominal full density.

**Figure 16:**
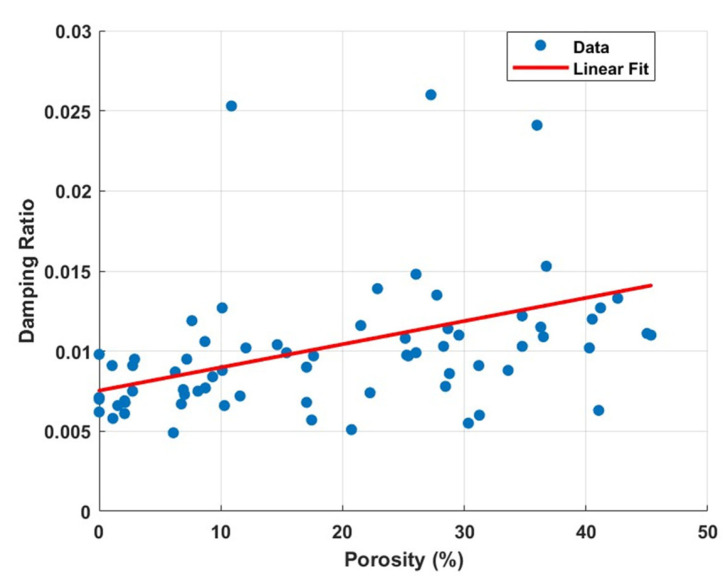
Scatter plot of porosity versus damping ratio for 3D-printed specimens.

Figure 16 illustrates the relationship between damping ratio and porosity in 3D-printed specimens. A linear regression model between porosity and damping ratio was fitted to the data, resulting in the following equation:

(23) ζ=0.0001P+0.0075

where ζ is the damping ratio and P is the porosity in percentage.

The model produced a low coefficient of determination (R^2^ = 0.166), indicating that only 16% of the variation in damping ratio can be explained by porosity alone. This implies that other factors play a more significant role in energy dissipation. Alternative modelling approaches, including higher-order polynomial and nonlinear regressions (e.g., second-order polynomial), were also evaluated but did not substantially improve the fit. This reinforces the conclusion that porosity, by itself, is not a strong predictor of damping behaviour within the tested range. Despite some data variability, a general trend is evident: higher porosity tends to be associated with slightly increased damping ratios [67]. This suggests that internal voids may enhance the structure’s capacity to dissipate vibrational energy. However, the presence of scattered data and outliers—particularly at the upper end of the damping ratio—may stem from experimental uncertainties or measurement errors. Moreover, the observed variability supports the notion that additional microstructural and process-dependent factors, such as infill pattern, inter-layer bonding, and material anisotropy, also influence damping behaviour and contribute to the overall response.

## 4. Conclusions

This study comprehensively investigated the dynamic behaviour of ABS cantilever beams produced by FDM, focusing on the effects of printing parameters and structural porosity. Using EMA and RSM, the findings reveal which process conditions significantly affect both natural frequency and damping ratio, offering insights into the vibrational performance of 3D-printed components.

Among the four investigated infill geometries, the gyroid pattern exhibited the highest natural frequencies at high infill densities, indicating a more robust and integrated internal structure. In contrast, the triangle pattern consistently delivered higher damping ratios, suggesting superior energy dissipation despite lower stiffness, while the cubic pattern showed intermediate behaviour. These contrasting trends can be attributed to differences in internal architecture: the cubic pattern offers a more uniform and continuous structure, leading to higher stiffness but limited energy dissipation, whereas the triangle pattern features angular intersections and discontinuous load paths that enhance internal friction and viscoelastic energy loss—especially when used with larger nozzle diameters that increase extrusion thickness and interlayer bonding.

A strong positive correlation was observed between infill density and natural frequency, as increased material volume led to higher stiffness. However, this also resulted in reduced damping ratios, likely due to the lower presence of internal voids and diminished energy dissipation pathways. This reflects a common trade-off between rigidity and damping in structural design.

The nozzle diameter was found to influence both stiffness and vibrational response by affecting extrusion volume. Larger nozzle diameters often increased natural frequencies and, in some cases, damping ratios. However, this effect was non-linear and dependent on the infill pattern used. This trend aligns with previous findings, which indicate that an increase in nozzle diameter—under constant layer thickness—can lead to higher flexural strength, tensile strength, and overall stiffness [45].

A general trend was observed between porosity and damping: as porosity increased, so did the damping ratio, indicating enhanced vibration energy absorption due to internal voids. However, this relationship was not strictly linear. Regression analyses showed limited correlation in some cases, implying that damping behaviour is also influenced by microstructural geometry, material anisotropy, and manufacturing precision.

Although slicers typically generate solid internal structures at 100% infill, small variations in toolpath and overlap settings across infill types led to measurable differences in mass and dynamic behaviour. These deviations, while minor, were evident in modal amplitude and damping measurements and were supported by porosity values approaching zero.

While the developed RSM models demonstrated high reliability for predicting natural frequencies (R^2^ > 0.85), their predictive accuracy for damping ratios was moderate. This limitation likely arises from the complex and nonlinear nature of damping mechanisms, which cannot be fully captured using polynomial-based models. Additionally, measurement noise in FRF data, environmental variability, and manufacturing inconsistencies such as interlayer adhesion and orientation may contribute to prediction errors. Recognizing these limitations, future studies may benefit from exploring advanced modelling techniques such as machine learning or hybrid physics-informed approaches to better capture the complex damping behaviour in FDM structures.

In conclusion, this research highlights the critical influence of infill pattern, density, nozzle size, raster angle, and porosity on the dynamic properties of FDM-manufactured structures. The findings provide a valuable framework for optimizing the vibrational performance of 3D-printed parts. In particular, careful selection of infill geometry is essential for applications requiring enhanced damping capacity, such as vibration isolation systems or mechanical energy absorbers. Based on the experimental findings, gyroid infill is recommended for applications requiring enhanced damping, particularly at intermediate infill densities (60–80%), where it exhibited up to 15–20% higher damping ratios compared to triangle or grid patterns. In contrast, triangle patterns consistently yielded higher natural frequencies and lower modal amplitudes, indicating superior stiffness. Designers may therefore consider using gyroid structures for passive vibration control, as well as triangle patterns where rigidity is prioritized.

Future work should incorporate microstructural or cross-sectional imaging to further validate filament bonding quality and void distribution. This study did not account for temperature-dependent material behaviour, surface roughness variations, or fatigue response under repeated loading. These factors are known to influence interlayer bonding quality and long-term energy dissipation. For instance, thermal gradients during printing can induce residual stresses; surface irregularities may alter local stiffness; and cyclic loading can progressively change damping characteristics. These aspects were beyond the scope of this work but are essential for future studies aiming at application in aerospace or mechanical systems with extended service lifetimes. Additionally, future work should incorporate image-based or CT-based porosity quantification for greater accuracy.

## Figures and Tables

**Table 1:** Various process parameters employed fabrication of specimens.

Parameter	Levels/Variations
Nozzle Size (mm)	0.4, 0.6, 0.8
Infill Density (%)	40, 60, 80, 100
Infill Pattern	Line, Gyroid, Cubic, Triangular
Raster Angles for Line Pattern (°)	0, ±45, 90

**Table 2:** Some mechanical properties of FDM ABS filament [26].

Parameter	Ultimaker^®^ ABS Red
Tensile modulus	1618.5 MPa
Tensile stress at yield	39.0 MPa
Tensile stress at break	33.9 MPa
Flexural modulus	2070.0 MPa
Flexural strength	70.5 MPa
Melting temperature Density	225–245 °C 1.1 g/cm^3^

**Table 3:** Natural frequencies and damping ratios of cubic, triangle, and gyroid infill patterns.

NS (mm)	ID (%)	Cubic (C)		Triangle (T)		Gyroid (G)	
		1st Frequency	Damping Ratio	1st Frequency	Damping Ratio	1st Frequency	Damping Ratio
0.4	40	16.543	0.0111	16.179	0.0110	15.126	0.0110
0.4	60	17.790	0.0088	17.775	0.0103	19.595	0.0091
0.4	80	19.823	0.0068	19.782	0.0102	19.684	0.0090
0.4	100	20.708	0.0067	21.581	0.0098	22.795	0.0076
0.6	40	18.111	0.0063	17.186	0.0102	16.989	0.0127
0.6	60	18.796	0.0060	18.378	0.0097	18.768	0.0110
0.6	80	20.789	0.0057	20.416	0.0088	21.289	0.0097
0.6	100	21.686	0.0095	21.803	0.0071	24.268	0.0077
0.8	40	18.448	0.0153	19.364	0.0122	18.004	0.0115
0.8	60	18.003	0.0135	19.339	0.0116	19.838	0.0108
0.8	80	19.790	0.0104	20.057	0.0106	21.883	0.0099
0.8	100	22.793	0.0075	22.192	0.0062	23.607	0.0087

NS: Nozzle size, ID: Infill density.

**Table 4:** Regression model statistics for dynamic properties of cubic infill pattern.

IP	Natural Frequencies					Damping Ratios				
	$R^{2}$	$R_{adj}^{2}$	$R_{pred}^{2}$	p Value	F Value	$R^{2}$	$R_{adj}^{2}$	$R_{pred}^{2}$	p Value	F Value
C_1_	0.924	0.86	0.554	0.00265	14.6	0.672	0.398	−0.875	0.1500	2.46
C_2_	0.977	0.94	0.630	0.00389	24.7	0.970	0.918	0.365	0.0067	18.5

IP: Infill pattern, C_1_: Quadratic model of cubic, C_2_: 3rd-order polynomial model of cubic.

**Table 5:** Regression model statistics for dynamic properties of triangle infill pattern.

IP	Natural Frequencies					Damping Ratios				
	$R^{2}$	$R_{adj}^{2}$	$R_{pred}^{2}$	p Value	F Value	$R^{2}$	$R_{adj}^{2}$	$R_{pred}^{2}$	p Value	F Value
**T_1_**	0.975	0.955	0.860	9.49 × 10^−5^	47.6	0.919	0.852	0.468	0.00317	13.6
**T_2_**	0.992	0.978	0.851	0.00052	69.5	0.984	0.956	0.738	0.00195	35.3

IP: Infill pattern, T_1_: Quadratic model of triangle, T_2_: 3rd-order polynomial model of triangle.

**Table 6:** Regression model statistics for dynamic properties of gyroid infill pattern.

IP	Natural Frequencies					Damping Ratios				
	$R^{2}$	$R_{adj}^{2}$	$R_{pred}^{2}$	p Value	F Value	$R^{2}$	$R_{adj}^{2}$	$R_{pred}^{2}$	p Value	F Value
**G_1_**	0.952	0.912	0.832	0.00068	23.8	0.922	0.856	0.642	0.00288	14.1
**G_2_**	0.959	0.887	0.263	0.0123	13.4	0.979	0.943	0.692	0.00331	26.8

IP: Infill pattern, G_1_: Quadratic model of gyroid, G_2_: 3rd-order polynomial model of gyroid.

**Table 7:** Regression model statistics for dynamic properties of line infill patterns.

IP	Natural Frequencies					Damping Ratios				
	$R^{2}$	$R_{adj}^{2}$	$R_{pred}^{2}$	p Value	F Value	$R^{2}$	$R_{adj}^{2}$	$R_{pred}^{2}$	p Value	F Value
**L**	0.892	0.855	0.801	2.14 × 10^−10^	23.9	0.772	0.693	0.565	2.35 × 10^−6^	9.77

IP: Infill pattern, L: Line pattern.

## Data Availability

The data presented in this study is available on request from the corresponding author.

## Appendix A

**Table A1:** Statistical data for different infill patterns.

Pattern	Component	Natural Frequencies			Damping Ratios		
		SumSq	DF	MeanSq	SumSq	DF	MeanSq
Cubic	Model	35.506	7	5.0723	0.00010297	7	1.4709 × 10^−5^
	Linear	31.669	2	15.834	3.9607 × 10^−5^	2	1.9804 × 10^−5^
	Nonlinear	3.8373	5	0.7675	6.3358 × 10^−5^	5	1.2672 × 10^−5^
	Residual	0.82285	4	0.2057	3.1814 × 10^−6^	4	7.9535 × 10^−7^
Triangle	Model	38.456	7	5.4938	3.2372 × 10^−5^	7	4.6246 × 10^−6^
	Linear	35.223	2	17.612	1.8211 × 10^−5^	2	9.1056 × 10^−6^
	Nonlinear	3.2332	5	0.64664	1.4161 × 10^−5^	5	2.8321 × 10^−6^
	Residual	0.31612	4	0.07903	5.2475 × 10^−7^	4	1.3119 × 10^−7^
Gyroid	Model	79.189	7	11.313	2.6133 × 10^−5^	7	3.7332 × 10^−6^
	Linear	77.974	2	38.987	2.3685 × 10^−5^	2	1.1843 × 10^−5^
	Nonlinear	1.2149	5	0.24298	2.4475 × 10^−6^	5	4.895 × 10^−7^
	Residual	3.3814	4	0.84535	5.565 × 10^−7^	4	1.3912 × 10^−7^
Line	Model	480.56	9	53.396	0.0012023	9	0.00013359
	Linear	418.85	3	139.62	0.00064058	3	0.00021353
	Nonlinear	61.712	6	10.285	0.00056173	6	9.3621 × 10^−5^
	Residual	57.985	26	2.2302	0.00035568	26	1.368 × 10^−5^
